# Identification of a partnership model between a university and not-for-profit organization to address health professions education and health inequality gaps through simulation-based education: A scoping review

**DOI:** 10.1371/journal.pone.0311349

**Published:** 2024-10-24

**Authors:** Samyah Siraj, Beheshta Momand, Ginny Brunton, Adam Dubrowski

**Affiliations:** Faculty of Health Sciences, Ontario Tech University, Oshawa, Ontario, Canada; Pontifical Catholic University of Rio de Janeiro: Pontificia Universidade Catolica do Rio de Janeiro, BRAZIL

## Abstract

Simulation-based education is a key aspect of health professions education used to aid healthcare providers in developing and maintaining clinical skills. Rural and remote healthcare providers have limited access to skills development opportunities. Training tools such as simulators are primarily limited to university and hospital-based research centers in urban areas. This scoping review aimed to examine current literature to identify a partnership model involving academic institutions and non-profit organizations (NPOs) that focuses on facilitating the wider distribution of simulators. The five-stage Arksey and O’Malley methodological framework for conducting scoping reviews and the Joanna Briggs Institute Manual for Evidence Synthesis was used to guide the scoping review. The search was conducted on five literature databases, three grey literature databases and through manual reference searching with an applied time frame of 2000 to 2022. The search identified 15 articles that met the eligibility criteria and were included in the study. Analysis of the articles revealed that no partnership model currently exists that facilitates the production and distribution of simulators through a partnership between academic institutions and NPOs. Establishing the partnership, acquiring funding, implementation, monitoring and evaluation, and dissemination were identified as key stages of a multi-institutional partnership. Further research is necessary to fill the gaps of the partnership process pertaining to the development and production of simulators to train healthcare providers.

## Introduction

Health professions education (HPE) is a field that recognizes the need for all healthcare providers to be competent and be able to develop their proficiency through access to optimal training resources [1]. Simulation plays a critical role in the context of HPE by providing learners with hands-on experiences in a safe environment. Rural and remote (R&R) healthcare providers in Canada and around the world, such as physicians and nurses, have limited access to simulation resources that allow them to develop and maintain skills compared to those in urban areas [2]. This can be due to reasons such as cost and distance from urban centers. The quality of care thereby may be partially impacted by this inequity in R&R communities [3]. The College of Family Physicians of Canada recognizes the importance of providers practicing in R&R settings to develop contextual competencies to adequately serve the needs of the communities they work in. To reform HPE for R&R providers, the College of Family Physicians of Canada highlights the need to develop tools, such as simulation technology, to improve access to clinical training and improve rural education programs [3].

A key aspect of HPE is simulation-based education (SBE), which is to replicate a real task or patient encounter for training or quality improvement purposes [4]. SBE serves as an optimal technique for assessment and evaluation within a controlled environment, enabling healthcare providers to enhance their clinical skills without jeopardizing patient safety [5]. The availability and access to simulation technology, such as simulators, in the R&R context may be limited by costs, logistics, and ethical considerations. As such, stakeholders such as regulatory bodies, program directors, researchers, innovators, and learners need to look for modern solutions to develop sustainable access to simulation.

Three-dimensional (3D) printing is an emerging field that promotes the improved availability of high quality simulators produced at a low cost, and developed using a flexible and sustainable manufacturing process [6]. However, barriers exist in distributing 3D printing technology to R&R settings, as it is currently limited primarily to universities and hospital-based research centers in urban areas [7, 8]. By using 3D printing technologies, simulators can be co-developed by researchers and end-point users (e.g., R&R doctors) in a research laboratory at a reduced cost, addressing HPE gaps for R&R healthcare providers [9].

When co-developing simulators in academic research laboratories, the development cost is expensed using research and development funding. This funding can cover the cost to designing, prototyping and conducting validity, acceptability, feasibility, and efficacy/effectiveness research [10, 11]. After the initial co-development and test of efficacy, the simulators need to be manufactured and supplied to healthcare institutions to train healthcare providers. However, no mechanism or partnership model currently exists to facilitate the process of moving simulators from research laboratories to the R&R healthcare sector. An example of this process is the partnership between maxSIMhealth and the Society of Rural Physicians of Canada (SRPC). MaxSIMhealth is a university research laboratory located at Ontario Tech University, Oshawa, Ontario, Canada, that works with the SRPC, a not-for-profit organization (NPO), to create sustainable simulators for healthcare provider training. The SRPC formerly used animal parts, car sponges, and similar models to train rural physicians during their Annual Rural and Remote Medicine Conference. This practice is deemed an unsustainable, unethical, and unstandardized approach to training. The SRPC partnered with maxSIMhealth to acquire simulators for workshops hosted at their 29^th^ and 30^th^ Medicine Course. Through this process, the SRPC became active creators of the solution by assisting with testing and evaluation. The process of this partnership started with maxSIMhealth providing the simulators for free during the first year of the partnership. The second year of the partnership, the SRPC purchased the simulators at cost and allowed for testing and evaluation of the simulators at the conference. For the 31^st^ Medicine Course, maxSIMhealth supplied the SRPC with simulators at cost through an agreement between the two organizations. The agreement allowed maxSIMhealth to retain the intellectual property (IP) of the simulators that have been collaboratively developed in return for supplying the simulators at cost for future conferences. It also allowed maxSIMhealth to use the IP for other purposes such as further research or commercialization.

Following research and development, once the solution is ready, academic institutions benefit by producing publications and presentations that can be used for future funding. However, their role in the partnership concludes after developing the solution. Although the academic institution can move forward and sell the simulators, this becomes disadvantageous for the NPO if purchased at commercial value as they contributed to developing the innovation. This creates the need to produce the simulator at a reduced cost to make it affordable to NPOs so that all partners can benefit from the partnership.

Partnerships and linkages are one of the top six priorities of public health systems research in Canada, which recognizes the need to establish partnerships between different sectors to improve public health system performance [12]. These sectors include educational institutions, healthcare providers, community-based organizations, and private sector organizations. Creating and mobilizing partnerships between university research laboratories, NPOs, and for-profit organizations (FPOs) can facilitate knowledge, IP and physical asset exchange, and improve capacity building within the public health system. Establishing a partnership to facilitate the process of manufacturing simulators in an academic research laboratory to distribute to hospitals and R&R healthcare institutions can improve healthcare provider training [13], in turn improving the health outcomes of the Canadian population.

For-profit models currently exist where entities can benefit and make a profit from commercializing the innovation developed in a research laboratory whereby the IP goes to a FPO or spin-off company to be sold commercially. The need to make a profit from this process defeats the purpose of producing affordable solutions and limits the use of such models for contexts that do not have the resources and funding to afford the innovation. Although mechanisms do exist for academic institutions to move IP from research laboratories to the market, the search within this scoping review only looked for partnership models between academic institutions and NPOs. As such, the purpose of this scoping review was to explore existing literature to identify if a partnership model exists that describes the process of university research laboratories collaborating with a NPO to deliver SBE solutions to the healthcare sector. General simulation technology was explored in this scoping review to broaden the scope of literature found, including but not limited to virtual reality, augmented reality, gaming, and additive manufacturing (AM). However, for the sake of illustrating the need of the partnership model identified, the discussion section of this paper only focuses on 3D-printing and AM techniques to develop the simulators, with the intent for the partnership model to be applicable or adaptable to all technologies.

## Methods

This scoping review was conducted following Arksey and O’Malley’s methodological framework for scoping studies [14]. The framework details five stages that were applied to this study: 1) identify the research question, 2) identify relevant studies, 3) select the studies, 4) chart the data, and 5) collate, summarize and report the results. The scoping review chapter of the Joanna Briggs Institute Manual for Evidence Synthesis and the Preferred Reporting Items for Systematic reviews and Meta-Analyses (PRISMA) extension for Scoping Reviews Checklist was used to guide and report the findings of this scoping review (S1 Checklist) [15, 16]. A protocol was published detailing the process of this scoping review and the search strategy, which was prepared in accordance with PRESS guidelines [17, 18]. Although FPOs were mentioned as a stakeholder in the scoping review protocol, for the purpose of adding clarity to the scoping review, they have been removed from this paper.

The objective of undertaking this scoping review was to examine current literature and identify an existing partnership model involving university research laboratories and NPOs focused on SBE. The research question explored through this scoping review was: What are the existing partnership models, or components of a model, that outline how a university research laboratory can collaborate with a NPO to deliver technological solutions to the healthcare education sector? The objectives of this scoping review were to identify existing partnership models that can be adapted to our context, and to identify strategies that can be applied to a SBE partnership.

### Search strategy

The search strategy was carried out on five literature databases: Ovid MEDLINE, PsycINFO, Scopus, Web of Science, and CINAHL. A combination of free-text codes and controlled subjecting headings were used to capture key concepts derived from the research question (see S1 Table). Limits were placed on the search engines for the article language, limiting it to English language articles, and for the time frame, focusing the search to articles published between 2000 to 2022. This time frame was chosen because simulation became an official area of scientific inquiry after 2000 to standardize its use in HPE with the aim of reducing the number of medical errors caused by healthcare providers [19]. Grey Matters (CADTH), OpenGrey, and Google Scholar were also searched to explore grey literature, in addition to a snowball search of relevant articles included in the study by manually searching the reference list.

### Article selection

**Fig 1:**
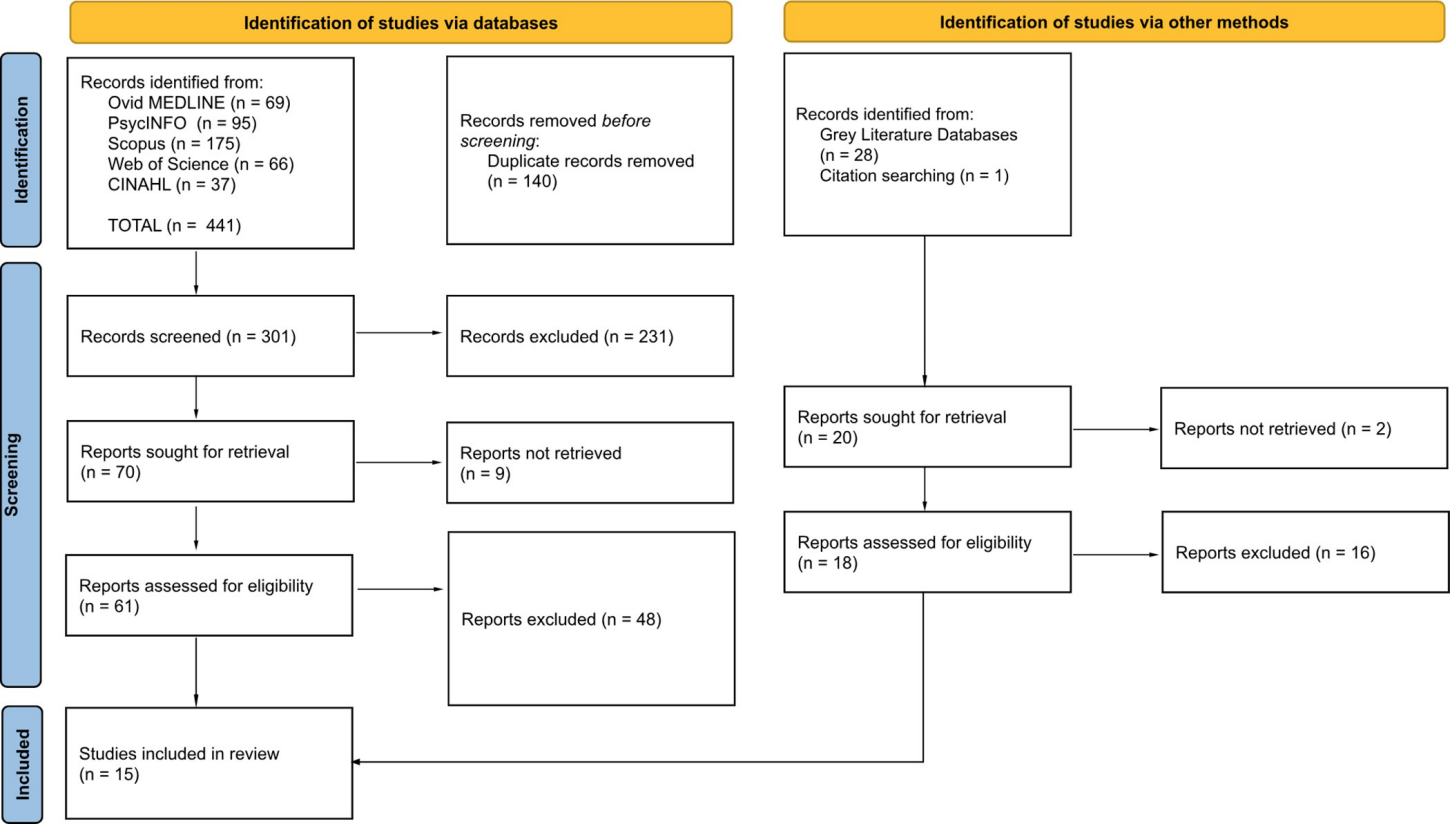
A PRISMA flow diagram of the search and screening process.

Articles were assessed using specific inclusion and exclusion criteria to select articles that addressed the research question. Articles were included if they described: 1) A partnership strategy, model, or framework, 2) involved both an academic institution and a non-profit organization, 3) focused on post-secondary education, and 4) discussed strategies that could apply to a SBE partnership. Articles were excluded if they focused on kindergarten to grade school, were published as an editorial, conference abstract, poster, or dissertation, and were not published in English. A two-step screening process was used to screen the articles. After the initial organization and removal of duplicate articles on Endnote 20, the refined search results were imported to a review software, EPPI-Reviewer. Articles were first screened by title and abstract to determine eligibility using the inclusion and exclusion criteria. This was followed by a full-text article screening where relevant articles were selected for the review. Fig 1 illustrates the screening process in a PRISMA flow diagram detailing the identification, screening, and inclusion of articles from the databases and other methods. Two reviewers (SS & BM) participated in step one of the screening process to review titles and abstracts. Both reviewers independently screened a subset of articles (30 articles) using the inclusion and exclusion criteria. Both reviewers met to discuss their ratings and refine the criteria, with disagreements resolved by consensus or a third reviewer. One reviewer (SS) then screened the remaining articles for both steps of the screening process to identify the relevant articles for data extraction.

### Data extraction and synthesis

One reviewer (SS) extracted and charted the data from all the studies and a second reviewer (BM) charted the data from a random sample of four articles to ensure charting quality. The data was extracted and charted on EPPI-Reviewer and the following information was extracted from the articles: publication year, country of origin, study design, participant characteristics, professions, study aims/objectives/purpose, technology developed, sentences discussing the partnership or collaboration model used, partnership strategies, and key findings related to the research question. Quantitative and qualitative data was summarized descriptively in text and presented using tables. The qualitative data was synthesized using themes and subthemes to align with the review objectives [20]. Potential models that can be adapted to the research context were reported along with characteristics of models that can be applied to a SBE partnership. Strategies that can be used to facilitate the partnership process have also been reported.

## Results

The search resulted in 441 articles being identified from the five published literature databases and 29 from other search methods. After removal of duplicates, 301 articles were screened by title and abstract and 79 articles were collectively assessed for eligibility at the full-text screening stage. A total of 15 articles passed eligibility screening and were included in the review.

### Study characteristics

The publication year for the 15 selected studies ranged from 2007 to 2022, with the majority (n = 11) published in the last 10 years. The country of origin for 11 of the included articles was the USA, among which three of the studies were conducted in collaboration with another country (Table 1) [22, 23, 26–33, 35]. A further two studies were conducted in Canada [24, 34], one in Bangladesh [21], and one in the Netherlands [25]. Case study was the most common study design identified (n = 3) [28, 32, 34], followed by multi-stakeholder dialogues (n = 1) [21], descriptive inquiry (n = 1) [24], review (n = 1) [25], mixed methods study (n = 1) [26], descriptive review (n = 1) [27], project report (n = 1) [29], and innovation report (n = 1) [33]. Five articles did not describe or were unclear on the study design [22, 23, 30, 31, 35].

**Table 1:** Characteristics of articles included in the study.

Author (Year)	Country of Origin	Study Design	Participants	Professions	What was the research intended to achieve?	Technology Developed	Partnership/ collaboration model used
Ashraf et al. (2015) [21]	Bangladesh	Multi- stakeholder dialogues (MSDs)	Industry, University/Academic, Community (NPO/NGO), Healthcare Institution, Government, Other	Researcher, Faculty/Professor, Government Leaders, Not described/Unclear	Increase stakeholder engagement in policy making and implementation (scale up) of a national Information and communication technologies or eHealth/mHealth strategy	Information and communication technologies (ICTs), eHealth/ mHealth	Not described/Unclear
Busse et al. (2013) [22]	USA and Ethiopia	Not described/ Unclear	University/Academic, Community (NPO/NGO), Healthcare Institution	Physician, Nurse, Faculty/Professor, Student/Resident	Describe the stages of a twinning partnership in the context of a collaboration between an American university and an Ethiopian-based university/hospital and NPO to strengthen emergency medicine in Ethiopia; understand if the twinning partnership was effective in training emergency medical professionals in Ethiopia	No technology/ simulation was developed or used in this partnership	Six-phase twinning partnership model
Cancedda et al. (2014) [23]	USA and Rwanda	Not described/ Unclear	University/Academic, Community (NPO/NGO), Healthcare Institution, Government	Physician, Nurse, Faculty/Professor, Student/Resident, Government Leaders, community health workers, allied health professionals	Strengthen formal educational and in-service training programs for local health professionals in Rwanda	No technology/ simulation was developed or used in this partnership	Health service delivery framework
De Civita & Dasgupta (2007) [24]	Canada	Descriptive inquiry	University/Academic, Healthcare Institution, Government	Physician, Nurse, Researcher, multidisciplinary diabetes management team, Project managers, decision makers	Re-examine the implementation experiences previously reported by the developers of a diabetes management pilot program in Montreal, focusing on identifying potentially important process factors that could effectively increase adoption and sustainability	A diabetes software/ computer system was used; No simulation was developed or used in this partnership	Diffusion of innovations theory
De Vrueh & Crommelin (2017) [25]	Netherlands	Review	Industry, University/Academic, Community (NPO/NGO), Healthcare Institution, Government, Other	Researcher, Not described/Unclear	Provide an understanding on the role of Public Private Partnerships (PPPs) in facilitating precompetitive multi-stakeholder collaborative research	No technology/ simulation was developed or used in this partnership	Multi-stakeholder collaborative research model/PPP model
Greece et al. (2019) [26]	USA	Mixed Methods Study	University/Academic, Community (NPO/NGO), Healthcare Institution	Researcher, Faculty/Professor, Public Health Agencies	Develop a practice-based teaching (PBT) framework to design, implement, and evaluate the PBT pedagogical approach with the intent to help prepare master of public health graduates for successful application of public health competencies in their careers	Learning management systems (LMS) software applications (not a primary component of the partnership)	PBT STEPS framework
Kerry et al. (2022) [27]	USA, Malawi, Tanzania, Uganda, Eswatini and Liberia	Descriptive Review	University/Academic, Community (NPO/NGO), Healthcare Institution, Government	Physician, Nurse, Faculty/Professor, Student/Resident, Government Leaders, Midwives	Help strengthen existing professional health education systems and care delivery by collaborating with partner countries to meet their immediate and long-term professional human resources for health needs	The establishment of simulation labs (not a primary component of the partnership)	Global health service partnership model
Liu et al. (2022) [28]	USA	Case Study	University/Academic, Community (NPO/NGO)	Researcher, community stakeholders	Describe a successful community-academic partnership, the process of collaboration and lessons learned from the partnership, which used a community-based participatory research (CBPR) approach	No technology/ simulation was developed or used in this partnership	Not described/Unclear
Magwood et al. (2012) [29]	USA	Project Report	University/Academic, Community (NPO/NGO), Government	Physician, Researcher, community partners, health services research methodologist, biostatistician	Activate a community of informed learners who are committed to the transformation and improvement of health outcomes for disparate communities	No technology/ simulation was developed or used in this partnership	Center for Community Health Partnership (CCHP) Model
Miller et al. (2012) [30]	USA	Not described/ Unclear	University/Academic, Community (NPO/NGO)	Researcher, community partners	Inform research-to-practice links for researchers looking to translate evidence-based programs (EBP) to community settings, and community-based organizations considering implementing an EBP	No technology/ simulation was developed or used in this partnership	Not described/Unclear
Olson et al. (2011) [31]	USA	Not described/ Unclear	Industry, University/Academic, Community (NPO/NGO), Healthcare Institution	Physician, Not described/Unclear	Describe a successful collaboration for continuing medical education	No technology/ simulation was developed or used in this partnership	Not described/Unclear
Payne (2014) [32]	USA	Case Study	Industry, University/Academic, Community (NPO/NGO), Healthcare Institution	Researcher, Not described/Unclear	Share the lessons learned using the Translational Research Informatics and Data	TRIAD Grid	Not described/Unclear
					Management (TRIAD) project example on how academic institutions and NPOs can license and commercialize technologies to achieve technology sustainability outside of traditional grants and contracts		
Taro et al. (2016) [33]	USA	Innovation Report	University/Academic, Community (NPO/NGO), Healthcare Institution	Physician, Surgeon, Researcher, Student/Resident	Share a process for global health partnerships and provide a basic model for interdisciplinary and international partnerships between academia and medical institutions	No technology/ simulation was developed or used in this partnership	Global Surgery Partnership
Yan et al. (2018) [34]	Canada	Case Study	University/Academic, Community (NPO/NGO), Government	Researcher, Other	Understand the role of NGOs in cross-sector social partnerships, specifically using the Poverty and Employment Precarity in Southern Ontario (PEPSO) Research Partnership as a case study	No technology/ simulation was developed or used in this partnership	Cross-sector social partnerships
Youn et al. (2019) [35]	USA	Not described/ Unclear	University/Academic, Community (NPO/NGO)	Researcher, mental health providers	Inform on the implementation strategies of a cognitive-behavioural theory program using a CBPR partnership framework between an academic institution and an NGO	No technology/ simulation was developed or used in this partnership	CBPR/ community-based implementation framework

### Participant characteristics

Participants in each study included a combination of industry, university/academic, community (NPO/non-governmental organization (NGO)), healthcare institution, government, and/or others. The combination of university/academic and community (NPO/NGO) was common across all 15 articles, with the addition of healthcare institutions in ten articles, government in seven articles, industry in four articles, and other organizations in two articles. The professions of individuals involved in the partnership varied in each article depending on the research being conducted and the type of partnership (see Table 1 for participants and professions for each article).

### Aims

Four articles shared a similar research aim, focused on increasing the training capacity through an in-service training program partnership between stakeholders [22, 23, 27, 33]. Two articles described the use of a university-community partnership for researchers to translate evidence-based programs to the community [30, 35]. A number of articles had similar purposes to describe a type of multi-stakeholder partnership within different contexts, such as 1) understanding the role of public-private partnerships (PPPs) to facilitate collaborative research [25], 2) describing a successful community-academic partnership to improve data management and analysis [28], 3) developing a community-academic partnership using community-based participatory research (CBPR) principles to improve community health [29], 4) describing a successful interorganizational collaboration for continuing medical education [31], and 5) understanding the role of non-governmental organizations (NGOs) in cross-sector social partnerships [34]. The remaining articles had unique research aims that did not overlap. One article focused on addressing eHealth implementation challenges through collaboration and knowledge exchange [21], while another article aimed to examine the use of diffusion of innovation theory (DIT) for a diabetes management program [24]. One article aimed to describe how to apply a practice-based teaching (PBT) framework [26], and one article intended to share lessons learned from the Translational Research Informatics and Data Management (TRIAD) project to commercialize a prototype technology [32].

### Key findings

Of the 15 articles selected for this scoping review, 10 articles had no technology or simulation component being studied or developed in the partnership [22, 23, 25, 28–31, 33–35]. Information and communication technologies (ICTs), and eHealth/mHealth was the technological focus of one study [21]. A diabetes software system was briefly mentioned in one study, and no simulation was developed or used in the partnership [24]. One study identified the use of a learning management system software application [26]. However, it was not a primary component of the partnership. Similarly, the establishment of simulation laboratories was listed as an outcome of a partnership but was not described in detail or was a focus of the partnership [27]. One article had a technological focus to the research, describing the development of the TRIAD Grid, a research-oriented data management and sharing infrastructure. However, no partnership model or framework was described in this article [32].

The partnership model/framework described in the articles fell within two categories: 1) Partnership models/frameworks, 2) General frameworks. Category one, partnership models/frameworks, described specific stages or steps within the model/framework that detailed a process to forming and conducting a partnership and/or strategies that can be used throughout the partnership. Category two, general frameworks, described theoretical or conceptual frameworks that were used to underpin the study and provided an understanding for the approach taken within the study. Articles with frameworks that fell within category two also provided strategies that can be used throughout the partnership process. Partnership models/frameworks that were described were the six-phase twinning partnership model [22], a multi-stakeholder PPP model [25], the PBT STEPS framework [26], the global health service partnership model [27], the center for community health partnership model [29], and the global surgery partnership [33]. No partnership model/framework overlapped, however, there was some similarity in the approach taken, with variations depending on the research context. General frameworks that were described were the health service delivery framework used to leverage training and research collaborations [23], the DIT framework used to understand the process of adopting novel technologies [24], cross-sector social partnerships as an underpinning process used to deal with complex social issues [34], and a community-based implementation framework emphasizing equitable partnerships between community members [35]. Principles from the CBPR framework were also highlighted as underpinning the research in three articles [28, 29, 35]. Five of the 15 articles did not describe any partnership model/framework that was used to guide the partnership [21, 28, 30–32].

No SBE partnership model was identified that outlined how a university research laboratory can collaborate with relevant stakeholders to deliver technological solutions to the healthcare education sector. Several partnership models/frameworks that were mentioned in the literature did not describe the process or stages of the model in-depth and did not have enough information to easily replicate the partnership process [21, 27, 29, 33–35]. The models/frameworks that did provide guidance on how to execute the partnership did not align with the context of the intended research [23, 25, 26].

One article described a partnership model that details stages relevant to the research context, the twinning partnership model [22]. The model details six stages of a twinning partnership between academic institutions and community organizations to collectively share resources and knowledge in a peer-to-peer relationship to produce technological and economical solutions. The six phases are: 1) initiate a partnership, 2) develop a shared work plan, 3) implement the program, 4) monitor outcomes, 5) evaluate results, and 6) disseminate information [22]. The twinning partnership model is useful in conducting partnerships between academic institutions and NPOs. The limitation of this model with regards to the identified research gap is the lack of a simulation component, and specifically simulation technology, as the central focus of the partnership. The model does not describe the process of manufacturing simulators and how to navigate issues that are essential to technology development, such as funding and IP, and ultimately disseminating the simulation technology to R&R settings.

Partnership strategies that can be used to facilitate the partnership were also explored. These strategies included what to do before, during and after the partnership. Several strategies described in the articles overlapped. After analysis of the literature, five themes of stages relevant to the partnership process, and 13 subthemes of strategies that can be applied in the different stages were revealed. Table 2 presents a detailed account of the subthemes categorized under each theme.

**Table 2:** Overview of themes and subthemes.

Themes	Subthemes	No. of Studies	Reference
**Engaging Partners/ Establishing the Partnership**	Building on existing relationships; Identifying appropriate stakeholders	7	[21–24, 26, 31, 33]
	Conducting a needs assessment	5	[22, 24, 26, 30, 35]
	Clear communication of, common goals, shared vision, benefits, and purpose	6	[22, 24, 25, 28, 31, 33]
	Clear division and definition of roles and responsibilities; expectation setting	8	[22–24, 26, 28, 30, 31, 33]
	Developing a shared work plan with clear objectives, goals, indicators/deliverables, outcomes, budget, and timeline; having a contract or memorandum of understanding (MOU)	9	[21, 22, 24–26, 28, 29, 31, 33]
**Acquiring Funding**	Acquiring funding from government, partnered organizations, grant programs, private donations, commercialization of technology	4	[23, 27, 28, 32]
**Implementation**	Open and frequent communication with partners through face-to-face meetings, conference calls, regular work plan meetings, emails; formal and informal communication; having a digital file sharing tool	7	[22, 24, 26, 28, 30, 31, 33]
	Involving key opinion leaders and clinical champions to support adoption of the partnership	2	[24, 31]
**Monitoring and Evaluation**	Use of a monitoring and evaluation framework; establishing a performance measurement system with measurable targets for each partner; formative and summative evaluation plan	4	[22, 25, 30, 31]
	Quality improvement evaluation for the process and outcome of the partnership	6	[22–24, 26, 30, 33]
	System for data collection and documentation; quantitative measures of productivity (activities, deliverables) collected using validated, quantitative reporting forms and qualitative measures of program outcomes assessed through a series of internal and external evaluations; feedback from partners	5	[22, 26, 27, 30, 35]
**Dissemination**	Communicating results with partners; sharing achievements and lessons learned	2	[22, 26]
	Strategic plan to disseminate results through reports, conference presentations	5	[22, 24, 26, 31, 35]

The stages that can be used for a SBE partnership that were derived from analysis of the articles are: 1) engaging partners/establishing the partnership, 2) acquiring funding, 3) implementation, 4) monitoring and evaluation, and 5) dissemination. These stages are considered fundamental to fulfilling a successful partnership in the healthcare sector. The first stage, engaging partners and establishing the partnership, is described as the process of communication with appropriate stakeholders and building the relationship to commence the partnership. This stage involves identifying the needs of each partner, communicating and aligning the vision and purpose of the partnership to benefit all partners, and dividing the roles and responsibilities for each partner with a clear work plan to guide the partnership activities [21–26, 28–31, 33, 35]. The second stage, acquiring funding, is necessary to actuate the partnership and requires a funding source or the collection of funds to carry out the objectives of the partnership [23, 27, 28, 32]. The implementation stage that follows requires consistent communication with partners to ensure the plan is on track and to hold the partners accountable [22, 24, 26, 28, 30, 31, 33]. This may involve the participation of opinion leaders and champions to support a successful implementation of the partnership [24–31]. The monitoring and evaluation stage is necessary to improve the process and outcomes of the partnership through an evaluation plan by collecting data using key indicators and feedback from partners [22–27, 30, 31, 33, 35]. The final stage, dissemination, is key to sharing the knowledge and lessons learned from the partnership to inform individuals who can utilize this knowledge within their own context [22, 24, 26, 31, 35]. The five stages provide a general process to carrying out a multi-institutional partnership. In addition, one article describes the process of an academic institution and NPO licensing and commercializing a technology. The article describes the process of moving technology from the research laboratory to the market in a climate where there is difficulty acquiring funding and grants for a project [32]. However, the article does not describe the process of manufacturing the technology and delivering it to the healthcare sector.

## Discussion

This scoping review is the first to examine the literature to identify a partnership model that focuses on the diffusion of simulators from research laboratories to community healthcare organizations, which is especially necessary in Canadian R&R settings. Answering the research question is significant as it pertains to finding a solution to HPE gaps for R&R healthcare providers. The overarching purpose of the articles included in the study was to improve community health outcomes, whether it be through improving health education programs or learning from previous multi-institutional partnerships. Physicians were the most common healthcare professionals identified in the articles, along with surgeons, nurses, residents, midwives, and/or other allied health professionals. These professionals were involved in projects that aimed to improve healthcare education and/or health outcomes [22–24, 27, 29, 31, 33]. The healthcare focus of individuals involved in the partnerships suggests a need to provide tailored training tools and resources. Few studies described international collaborations between two or more countries. Where this did occur, they were primarily between resource-rich and low-income countries, highlighting the importance of knowledge and resource exchange to address disparities in healthcare training and service delivery [22, 23, 27].

We identified no existing partnership model that involved university research laboratories and NPOs where the aim was to deliver simulation solutions. Of those identified, the model that came closest to describing a partnership process that could be useful in developing a SBE partnership model was the twinning partnership model. However, this model lacks a simulation component and consequently the necessary details to manufacture and facilitate the process of distributing simulators to R&R areas [22]. The articles provided relevant information that can be used to guide the process from the beginning to end of a SBE partnership. The five themes and 13 subthemes derived from the literature identify stages and strategies that can be used as a template to structure the process of a SBE partnership. The information and process needs to be further contextualized to fit the research context and add the necessary elements to detail the process of manufacturing and distributing the simulators.

### Principles of the partnership

A successful multi-institutional partnership in the healthcare context has been identified as being guided by key principles that are rooted in integrated knowledge translation (iKT) and CBPR. Several articles discussed iKT and CBPR principles as an integral part to executing a successful partnership. These principles include producing mutually beneficial solutions, grounding the partnership in shared principles, building long-term relationships, and involving all partners in the decision-making process to establish a shared vision, co-learning, co-ownership, and co-leadership. In addition, it is important to establish mutual respect, trust, and understanding, prioritize reciprocity and bilateral innovation, and allow partners to take an active role in supporting the creation of new knowledge [22–25, 28–31, 35]. These guiding principles are key to producing and enhancing research findings that are directly related to the knowledge users (i.e., healthcare providers and administrators). This collaborative approach to generating knowledge is an ideal way to address complex healthcare issues and improve healthcare service delivery[36].

### Gaps in the research

In relation to a SBE partnership model aiming to diffuse simulators from university research laboratories to hospitals and NPOs, existing models have several gaps in the purpose and process of the partnership. The identified models do not focus on simulation technology being the purpose of forming the partnership and primarily discuss the overarching process without examining the various stages of the partnership in detail. Due to the lack of a simulation technology component, the research and development process of the simulators is not described. The articles that do include a type of simulation technology do not use a physical training model and therefore do not include any information on the manufacturing process. In addition, few articles briefly discuss the sources of funding for their project, however, the process of acquiring funding to expense the production of the simulators or for general project management was not discussed. As such, further research needs to be conducted to fill in the identified gaps and create a new model that incorporates the dissemination of simulation technology to the healthcare sector to address training gaps in HPE [10, 14]. A new partnership model can be created by combining general partnership stages and strategies identified from this scoping review with findings collected from future research initiatives involving key stakeholders. Future research directions will involve developing a partnership model that details the partnership process of developing simulators in a research laboratory and partnering with FPOs and NPOs to manufacture and distribute the simulators to healthcare providers to improve rural medical education.

### Specific limitations

The purpose of this scoping review was to identify an existing partnership model that detailed the collaboration between academic institutions and NPOs to deliver simulation solutions to the healthcare sector. The limitations in this review are largely a result of AM being an emerging field, with 3D printing being the most common form, and consequently limited publications available on the diffusion of simulation technologies. Thirteen studies that were included in the scoping review were conducted outside of Canada. As such, the information presented in the articles do not entirely apply to Canadian partnerships and need to be adapted to fit the Canadian context. The scoping review revealed that most identified partnership models were situated in low- and middle-income countries (LMIC). While this was not the initial focus, and the partnership example of maxSIMhealth and SRPC was centered on R&R Canada rather than a LMIC, the scarcity of literature on developed countries emphasizes the necessity of contextualizing the review findings and devising a solution tailored to the Canadian R&R context. The prevalence of LMIC-based models underscores the need for a nuanced approach in addressing partnerships in resource-rich environments. Non-English articles were also excluded from the review, reducing the scope of literature that addresses the research question.

## Conclusion

SBE is a key component of HPE used to provide training to healthcare providers and is crucial in increasing the training capacity of R&R healthcare providers. The results of this scoping review identify a clear gap in the literature pertaining to the existence of a SBE partnership model. Currently, no model exists that facilitates a partnership between academic institutions and NPOs to produce simulators for healthcare provider training. The twinning partnership model is the most comparable model that can provide detail on general partnership stages that can be integrated into a SBE partnership model involving the appropriate stakeholders. Several components and partnership strategies used in existing models have been identified that can be incorporated into a SBE partnership model. Further research is necessary to identify the process of research and development, and manufacturing of the simulators to incorporate the process into a SBE partnership model. This will require collaborative research with key stakeholders involved in the partnership process to inform the creation of a new partnership model that will be used to deliver simulation solutions to R&R healthcare settings.

## Supporting information

S1 Checklist Filled PRISMA-ScR checklist. (DOCX)

S1 Table Search strategy performed on Ovid MEDLINE. (DOCX)

S1 Dataset Database search string and results. (DOCX)

S2 Dataset Identified studies and exclusions. (XLSX)

S3 Dataset Charted data. (XLSX)
