# Supplementary material for: Identification of a partnership model between a university and not-for-profit organization to address health professions education and health inequality gaps through simulation-based education: A scoping review
Source: PLoS One. 2024 Oct 24;19(10):e0311349. doi: 10.1371/journal.pone.0311349 (PMC11500885; doi:10.1371/journal.pone.0311349)
Supplement: S1 Table — (DOCX) [file pone.0311349.s002.docx]

**Search Strategy performed on Ovid MEDLINE**

| **Line #** | **Code** |
| --- | --- |
| **1** | exp Universities/ |
| **2** | exp *"Academies and Institutes"/ |
| **3** | exp Students/ |
| **4** | "academic institut*".mp. |
| **5** | "research institut*".mp. |
| **6** | universit*.mp. |
| **7** | colleg*.mp. |
| **8** | "post-secondary".mp. |
| **9** | “Post secondary”.mp. |
| **10** | 1 OR 2 OR 3 OR 4 OR 5 OR 6 OR 7 OR 8 OR 9 |
| **11** | exp Partnership Practice/ |
| **12** | exp Models, Organizational/ |
| **13** | exp Interinstitutional Relations/ |
| **14** | exp Cooperative Behavior/ |
| **15** | exp Public-Private Sector Partnerships/ |
| **16** | exp Intersectoral Collaboration/ |
| **17** | collaborat*.mp. |
| **18** | partnership*.mp. |
| **19** | "partnership model*".mp. |
| **20** | "partnership framework*".mp. |
| **21** | "collaborat* model*".mp. |
| **22** | "model* for collaboration".mp. |
| **23** | "model* for partnership*".mp. |
| **24** | "partnership* for collaboration".mp. |
| **25** | "collaborat* framework*".mp. |

| **26** | 11 OR 12 OR 13 OR 14 OR 15 OR 16 OR 17 OR 18 OR 19 OR 20 OR 21 OR 22  OR 23 OR 24 OR 25 |
| --- | --- |
| **27** | exp Organizations, Nonprofit/ |
| **28** | "non profit*".mp. |
| **29** | "non government*".mp. |
| **30** | nonprofit*.mp. |
| **31** | nongovernment*.mp. |
| **32** | 27 OR 28 OR 29 OR 30 OR 31 |
| **33** | exp "Delivery of Health Care"/ |
| **34** | exp Hospitals/ |
| **35** | healthcare.mp. |
| **36** | "health care".mp. |
| **37** | hospital.mp. |
| **38** | 33 OR 34 OR 35 OR 36 OR 37 |
| **39** | exp technology/ |
| **40** | exp printing, three-dimensional/ |
| **41** | exp Simulation Training/ |
| **42** | exp High Fidelity Simulation Training/ |
| **43** | exp "diffusion of innovation"/ |
| **44** | technolog*.mp. |
| **45** | simulat*.mp. |
| **46** | "simulation-based education".mp. |
| **47** | innovat*.mp. |
| **48** | 39 OR 40 OR 41 OR 42 OR 43 OR 44 OR 45 OR 46 OR 47 |
| **49** | 10 AND 26 AND 32 AND 38 AND 48 |
| **50** | limit 49 to (english language and yr="2000 -Current") |
