# Supplementary material for: Identification of a partnership model between a university and not-for-profit organization to address health professions education and health inequality gaps through simulation-based education: A scoping review
Source: PLoS One. 2024 Oct 24;19(10):e0311349. doi: 10.1371/journal.pone.0311349 (PMC11500885; doi:10.1371/journal.pone.0311349)
Supplement: S1 Dataset — (DOCX) [file pone.0311349.s003.docx]

**Ovid MEDLINE**

| **#** | **Query** | **Results from 6 Feb 2023** |
| --- | --- | --- |
| 1 | exp Universities/ | 51,281 |
| 2 | exp *"Academies and Institutes"/ | 19,060 |
| 3 | exp Students/ | 162,862 |
| 4 | "academic institut*".mp. | 7,923 |
| 5 | "research institut*".mp. | 13,468 |
| 6 | universit*.mp. | 489,334 |
| 7 | colleg*.mp. | 153,050 |
| 8 | "postsecondary".mp. | 1,305 |
| 9 | "Post secondary".mp. | 1,133 |
| 10 | 1 or 2 or 3 or 4 or 5 or 6 or 7 or 8 or 9 | 748,188 |
| 11 | exp Partnership Practice/ | 1,332 |
| 12 | exp Models, Organizational/ | 19,543 |
| 13 | exp Interinstitutional Relations/ | 10,827 |
| 14 | exp Cooperative Behavior/ | 45,859 |
| 15 | exp Public-Private Sector Partnerships/ | 2,468 |
| 16 | exp Intersectoral Collaboration/ | 2,569 |
| 17 | collaborat*.mp. | 193,870 |
| 18 | partnership*.mp. | 47,969 |
| 19 | "partnership model*".mp. | 585 |
| 20 | "partnership framework*".mp. | 35 |
| 21 | "collaborat* model*".mp. | 1,251 |
| 22 | "model* for collaboration".mp. | 430 |
| 23 | "model* for partnership*".mp. | 159 |
| 24 | "partnership* for collaboration".mp. | 200 |
| 25 | "collaborat* framework*".mp. | 238 |
| 26 | 11 or 12 or 13 or 14 or 15 or 16 or 17 or 18 or 19 or 20 or 21 or 22 or 23 or 24 or 25 | 280,975 |
| 27 | exp Organizations, Nonprofit/ | 20,427 |
| 28 | "non profit*".mp. | 2,983 |
| 29 | "non government*".mp. | 6,070 |
| 30 | nonprofit*.mp. | 7,346 |
| 31 | nongovernment*.mp. | 3,725 |
| 32 | 27 or 28 or 29 or 30 or 31 | 36,158 |
| 33 | exp "Delivery of Health Care"/ | 1,208,150 |
| 34 | exp Hospitals/ | 312,513 |
| 35 | [healthcare.mp](http://healthcare.mp/). | 353,277 |
| 36 | "health care".mp. | 915,472 |
| 37 | [hospital.mp](http://hospital.mp/). | 1,477,829 |
| 38 | 33 or 34 or 35 or 36 or 37 | 3,127,610 |
| 39 | exp technology/ | 488,452 |
| 40 | exp printing, three-dimensional/ | 11,226 |
| 41 | exp Simulation Training/ | 11,433 |
| 42 | exp High Fidelity Simulation Training/ | 373 |
| 43 | exp "diffusion of innovation"/ | 21,422 |
| 44 | technolog*.mp. | 712,564 |
| 45 | simulat*.mp. | 817,844 |
| 46 | "simulation-based education".mp. | 787 |
| 47 | innovat*.mp. | 220,843 |
| 48 | 39 or 40 or 41 or 42 or 43 or 44 or 45 or 46 or 47 | 2,012,181 |
| 49 | 10 and 26 and 32 and 38 and 48 | 75 |
| 50 | limit 49 to (english language and yr="2000 -Current") | 69 |

**PsycINFO**

| S51 | ("academic institut*" OR "research institut*" OR MAINSUBJECT.EXACT.EXPLODE("Research and Development") OR universit* OR MAINSUBJECT.EXACT.EXPLODE("Education") OR MAINSUBJECT.EXACT.EXPLODE("Schools") OR MAINSUBJECT.EXACT.EXPLODE("Academic Settings") OR MAINSUBJECT.EXACT.EXPLODE("Colleges") OR colleg* OR MAINSUBJECT.EXACT("Postgraduate Students") OR "Post secondary" OR MAINSUBJECT.EXACT.EXPLODE("Higher Education") OR postsecondary) AND (MAINSUBJECT.EXACT.EXPLODE("Collaboration") OR collaborat* OR partnership* OR ("partnership models") OR ("partnership framework") OR MAINSUBJECT.EXACT("Collaborative Learning") OR "collaborat* model*" OR "model* for collaboration" OR "model* for partnership*" OR MAINSUBJECT.EXACT("Cooperation") OR "partnership* for collaboration" OR "collaborat* framework*") AND (MAINSUBJECT.EXACT("Nonprofit Organizations") OR MAINSUBJECT.EXACT("NGOs") OR ("non government" OR "non governmental") OR nonprofit* OR nongovernment* OR "non profit*") AND (MAINSUBJECT.EXACT.EXPLODE("Health Care Services") OR MAINSUBJECT.EXACT.EXPLODE("Health Care Delivery") OR healthcare OR "health care" OR MAINSUBJECT.EXACT("Hospitals") OR hospital OR hospitals) AND (MAINSUBJECT.EXACT.EXPLODE("Technology") OR technolog* OR MAINSUBJECT.EXACT.EXPLODE("Simulation") OR simulat* OR "simulation-based education" OR MAINSUBJECT.EXACT("Innovation") OR innovat*) | 95 | |
| --- | --- | --- | --- |
| S50 | MAINSUBJECT.EXACT.EXPLODE("Technology") OR technolog* OR MAINSUBJECT.EXACT.EXPLODE("Simulation") OR simulat* OR "simulation-based education" OR MAINSUBJECT.EXACT("Innovation") OR innovat* | 606888 | |
| S49 | innovat* | 103805 | |
| S48 | MAINSUBJECT.EXACT("Innovation") | 16984 | |
| S47 | "simulation-based education" | 83 | |
| S46 | simulat* | 99129 | |
| S45 | MAINSUBJECT.EXACT.EXPLODE("Simulation") | 78855 | |
| S44 | technolog* | 285137 | |
| S43 | MAINSUBJECT.EXACT.EXPLODE("Technology") | 267365 | |
| S42 | MAINSUBJECT.EXACT.EXPLODE("Health Care Services") OR MAINSUBJECT.EXACT.EXPLODE("Health Care Delivery") OR healthcare OR "health care" OR MAINSUBJECT.EXACT("Hospitals") OR hospital OR hospitals | 860717 | |
| S41 | hospitals | 479036 | |
| S40 | hospital | 479036 | |
| S39 | MAINSUBJECT.EXACT("Hospitals") | 18858 | |
| S38 | "health care" | 277455 | |
| S37 | healthcare | 171913 | |
| S36 | MAINSUBJECT.EXACT.EXPLODE("Health Care Delivery") | 111605 | |
| S35 | MAINSUBJECT.EXACT.EXPLODE("Health Care Services") | 256862 | |
| S34 | MAINSUBJECT.EXACT("Nonprofit Organizations") OR MAINSUBJECT.EXACT("NGOs") OR ("non government" OR "non governmental") OR nonprofit* OR nongovernment* OR "non profit*" | 13653 | |
| S33 | "non profit*" | 2770 | |
| S32 | nongovernment* | 2068 | |
| S31 | nonprofit* | 7353 | |
| S30 | ("non government" OR "non governmental") | 2719 | |
| S29 | MAINSUBJECT.EXACT("NGOs") | 1544 | |
| S28 | MAINSUBJECT.EXACT("Nonprofit Organizations") | 2998 | |
| S27 | MAINSUBJECT.EXACT.EXPLODE("Collaboration") OR collaborat* OR partnership* OR ("partnership models") OR ("partnership framework") OR MAINSUBJECT.EXACT("Collaborative Learning") OR "collaborat* model*" OR "model* for collaboration" OR "model* for partnership*" OR MAINSUBJECT.EXACT("Cooperation") OR "partnership* for collaboration" OR "collaborat* framework*" | 149441 | |
| S26 | "collaborat* framework*" | 115 | |
| S25 | "partnership* for collaboration" | 5 | |
| S24 | MAINSUBJECT.EXACT("Cooperation") | 16244 | |
| S23 | "model* for partnership*" | 13 | |
| S22 | "model* for collaboration" | 64 | |
| S21 | "collaborat* model*" | 921 | |
| S20 | MAINSUBJECT.EXACT("Collaborative Learning") | 4303 | |
| S19 | ("partnership framework") | 21 | |
| S18 | ("partnership models") | 81 | |
| S17 | partnership* | 34294 | |
| S16 | collaborat* | 111892 | |
| S15 | MAINSUBJECT.EXACT.EXPLODE("Collaboration") | 14764 | |
| S14 | "academic institut*" OR "research institut*" OR MAINSUBJECT.EXACT.EXPLODE("Research and Development") OR universit* OR MAINSUBJECT.EXACT.EXPLODE("Education") OR MAINSUBJECT.EXACT.EXPLODE("Schools") OR MAINSUBJECT.EXACT.EXPLODE("Academic Settings") OR MAINSUBJECT.EXACT.EXPLODE("Colleges") OR colleg* OR MAINSUBJECT.EXACT("Postgraduate Students") OR "Post secondary" OR MAINSUBJECT.EXACT.EXPLODE("Higher Education") OR postsecondary | 3236763 | |
| S13 | postsecondary | 6790 | |
| S12 | MAINSUBJECT.EXACT.EXPLODE("Higher Education") | 64165 | |
| S11 | "Post secondary" | 3420 | |
| S10 | MAINSUBJECT.EXACT("Postgraduate Students") | 1800 | |
| S9 | colleg* | 675251 | |
| S8 | MAINSUBJECT.EXACT.EXPLODE("Colleges") | 19117 | |
| S7 | MAINSUBJECT.EXACT.EXPLODE("Academic Settings") | 80647 | |
| S6 | MAINSUBJECT.EXACT.EXPLODE("Schools") | 79242 | |
| S5 | MAINSUBJECT.EXACT.EXPLODE("Education") | 478820 | |
| S4 | universit* | 2842370 | |
| S3 | MAINSUBJECT.EXACT.EXPLODE("Research and Development") | 33231 | |
| S2 | "research institut*" | 73751 | |
| S1 | "academic institut*" | 1924 | |

**Scopus**

| 1 | TITLE-ABS-KEY ( "academic institut*" ) | 17,505 |
| --- | --- | --- |
| 2 | TITLE-ABS-KEY ( "research institut*" ) | 79,395 |
| 3 | TITLE-ABS-KEY ( universit* ) | 1,829,844 |
| 4 | TITLE-ABS-KEY ( colleg* ) | 513,978 |
| 5 | TITLE-ABS-KEY ( "post secondary" ) | 5,731 |
| 6 | TITLE-ABS-KEY ( “higher education” ) | 181,820 |
| 7 | TITLE-ABS-KEY ( academi* ) | 1,032,677 |
| 8 | TITLE-ABS-KEY ( postsecondary ) | 7,478 |
| 9 | TITLE-ABS-KEY ( student* ) | 1,477,029 |
| 10 | ( TITLE-ABS-KEY ( "academic institut*" ) ) OR ( TITLE-ABS-KEY ( "research institut*" ) ) OR ( TITLE-ABS-KEY ( universit* ) ) OR ( TITLE-ABS-KEY ( colleg* ) ) OR ( TITLE-ABS-KEY ( "post secondary" ) ) OR ( TITLE-ABS-KEY ( "higher education" ) ) OR ( TITLE-ABS-KEY ( academi* ) ) OR ( TITLE-ABS-KEY ( postsecondary ) ) OR ( TITLE-ABS-KEY ( student* ) ) | 4,069,33 |
| 11 | TITLE-ABS-KEY ( collaborat* ) | 692,253 |
| 12 | TITLE-ABS-KEY ( partnership* ) | 165,328 |
| 13 | TITLE-ABS-KEY ( "partnership model*" ) | 1,777 |
| 14 | TITLE-ABS-KEY ( "partnership framework*" ) | 169 |
| 15 | TITLE-ABS-KEY ( "collaborat* model*" ) | 5,049 |
| 16 | TITLE-ABS-KEY ( "model* for collaboration" ) | 311 |
| 17 | TITLE-ABS-KEY ( "model* for partnership*" ) | 52 |
| 18 | TITLE-ABS-KEY ( "partnership* for collaboration" ) | 6 |
| 19 | TITLE-ABS-KEY ( "collaborat* framework*" ) | 1,765 |
| 20 | TITLE-ABS-KEY ( cooperati* ) | 714,801 |
| 21 | ( TITLE-ABS-KEY ( collaborat* ) ) OR ( TITLE-ABS-KEY ( partnership* ) ) OR ( TITLE-ABS-KEY ( "partnership model*" ) ) OR ( TITLE-ABS-KEY ( "partnership framework*" ) ) OR ( TITLE-ABS-KEY ( "collaborat* model*" ) ) OR ( TITLE-ABS-KEY ( "model* for collaboration" ) ) OR ( TITLE-ABS-KEY ( "model* for partnership*" ) ) OR ( TITLE-ABS-KEY ( "partnership* for collaboration" ) ) OR ( TITLE-ABS-KEY ( "collaborat* framework*" ) ) OR ( TITLE-ABS-KEY ( cooperati* ) ) | 1,457,973 |
| 22 | TITLE-ABS-KEY ( "non profit*" ) | 31,178 |
| 23 | TITLE-ABS-KEY ( "non government*" ) | 26,080 |
| 24 | TITLE-ABS-KEY ( nonprofit* ) | 21,545 |
| 25 | TITLE-ABS-KEY ( nongovernment* ) | 16,345 |
| 26 | ( TITLE-ABS-KEY ( "non profit*" ) ) OR ( TITLE-ABS-KEY ( "non government*" ) ) OR ( TITLE-ABS-KEY ( nonprofit* ) ) OR ( TITLE-ABS-KEY ( nongovernment* ) ) | 84,632 |
| 27 | TITLE-ABS-KEY ( healthcare ) | 530,580 |
| 28 | TITLE-ABS-KEY ( "health care" ) | 1,992,945 |
| 29 | TITLE-ABS-KEY ( hospital ) | 2,408,481 |
| 30 | ( TITLE-ABS-KEY ( healthcare ) ) OR ( TITLE-ABS-KEY ( "health care" ) ) OR ( TITLE-ABS-KEY ( hospital ) ) | 4,163,006 |
| 31 | TITLE-ABS-KEY ( technolog* ) | 4,267,353 |
| 32 | TITLE-ABS-KEY ( simulat* ) | 5,516,958 |
| 33 | TITLE-ABS-KEY ( "simulation based education" ) | 929 |
| 34 | TITLE-ABS-KEY ( innovat* ) | 990,686 |
| 35 | TITLE-ABS-KEY ( “three dimensional printing” ) | 21,199 |
| 36 | ( TITLE-ABS-KEY ( technolog* ) ) OR ( TITLE-ABS-KEY ( simulat* ) ) OR ( TITLE-ABS-KEY ( "simulation-based education" ) ) OR ( TITLE-ABS-KEY ( innovat* ) ) OR ( TITLE-ABS-KEY ( "three dimensional printing" ) ) | 9,994,461 |
| 37 | ( ( TITLE-ABS-KEY ( "academic institut*" ) ) OR ( TITLE-ABS-KEY ( "research institut*" ) ) OR ( TITLE-ABS-KEY ( universit* ) ) OR ( TITLE-ABS-KEY ( colleg* ) ) OR ( TITLE-ABS-KEY ( "post secondary" ) ) OR ( TITLE-ABS-KEY ( "higher education" ) ) OR ( TITLE-ABS-KEY ( academi* ) ) OR ( TITLE-ABS-KEY ( postsecondary ) ) OR ( TITLE-ABS-KEY ( student* ) ) ) AND ( ( TITLE-ABS-KEY ( collaborat* ) ) OR ( TITLE-ABS-KEY ( partnership* ) ) OR ( TITLE-ABS-KEY ( "partnership model*" ) ) OR ( TITLE-ABS-KEY ( "partnership framework*" ) ) OR ( TITLE-ABS-KEY ( "collaborat* model*" ) ) OR ( TITLE-ABS-KEY ( "model* for collaboration" ) ) OR ( TITLE-ABS-KEY ( "model* for partnership*" ) ) OR ( TITLE-ABS-KEY ( "partnership* for collaboration" ) ) OR ( TITLE-ABS-KEY ( "collaborat* framework*" ) ) OR ( TITLE-ABS-KEY ( cooperati* ) ) ) AND ( ( TITLE-ABS-KEY ( "non profit*" ) ) OR ( TITLE-ABS-KEY ( "non government*" ) ) OR ( TITLE-ABS-KEY ( nonprofit* ) ) OR ( TITLE-ABS-KEY ( nongovernment* ) ) ) AND ( ( TITLE-ABS-KEY ( healthcare ) ) OR ( TITLE-ABS-KEY ( "health care" ) ) OR ( TITLE-ABS-KEY ( hospital ) ) ) AND ( ( TITLE-ABS-KEY ( technolog* ) ) OR ( TITLE-ABS-KEY ( simulat* ) ) OR ( TITLE-ABS-KEY ( "simulation-based education" ) ) OR ( TITLE-ABS-KEY ( innovat* ) ) OR ( TITLE-ABS-KEY ( "three dimensional printing" ) ) ) | 199 |
| 38 | ( ( TITLE-ABS-KEY ( "academic institut*" ) ) OR ( TITLE-ABS-KEY ( "research institut*" ) ) OR ( TITLE-ABS-KEY ( universit* ) ) OR ( TITLE-ABS-KEY ( colleg* ) ) OR ( TITLE-ABS-KEY ( "post secondary" ) ) OR ( TITLE-ABS-KEY ( "higher education" ) ) OR ( TITLE-ABS-KEY ( academi* ) ) OR ( TITLE-ABS-KEY ( postsecondary ) ) OR ( TITLE-ABS-KEY ( student* ) ) ) AND ( ( TITLE-ABS-KEY ( collaborat* ) ) OR ( TITLE-ABS-KEY ( partnership* ) ) OR ( TITLE-ABS-KEY ( "partnership model*" ) ) OR ( TITLE-ABS-KEY ( "partnership framework*" ) ) OR ( TITLE-ABS-KEY ( "collaborat* model*" ) ) OR ( TITLE-ABS-KEY ( "model* for collaboration" ) ) OR ( TITLE-ABS-KEY ( "model* for partnership*" ) ) OR ( TITLE-ABS-KEY ( "partnership* for collaboration" ) ) OR ( TITLE-ABS-KEY ( "collaborat* framework*" ) ) OR ( TITLE-ABS-KEY ( cooperati* ) ) ) AND ( ( TITLE-ABS-KEY ( "non profit*" ) ) OR ( TITLE-ABS-KEY ( "non government*" ) ) OR ( TITLE-ABS-KEY ( nonprofit* ) ) OR ( TITLE-ABS-KEY ( nongovernment* ) ) ) AND ( ( TITLE-ABS-KEY ( healthcare ) ) OR ( TITLE-ABS-KEY ( "health care" ) ) OR ( TITLE-ABS-KEY ( hospital ) ) ) AND ( ( TITLE-ABS-KEY ( technolog* ) ) OR ( TITLE-ABS-KEY ( simulat* ) ) OR ( TITLE-ABS-KEY ( "simulation-based education" ) ) OR ( TITLE-ABS-KEY ( innovat* ) ) OR ( TITLE-ABS-KEY ( "three dimensional printing" ) ) ) AND ( LIMIT-TO ( PUBYEAR , 2022 ) OR LIMIT-TO ( PUBYEAR , 2021 ) OR LIMIT-TO ( PUBYEAR , 2020 ) OR LIMIT-TO ( PUBYEAR , 2019 ) OR LIMIT-TO ( PUBYEAR , 2018 ) OR LIMIT-TO ( PUBYEAR , 2017 ) OR LIMIT-TO ( PUBYEAR , 2016 ) OR LIMIT-TO ( PUBYEAR , 2015 ) OR LIMIT-TO ( PUBYEAR , 2014 ) OR LIMIT-TO ( PUBYEAR , 2013 ) OR LIMIT-TO ( PUBYEAR , 2012 ) OR LIMIT-TO ( PUBYEAR , 2011 ) OR LIMIT-TO ( PUBYEAR , 2010 ) OR LIMIT-TO ( PUBYEAR , 2009 ) OR LIMIT-TO ( PUBYEAR , 2008 ) OR LIMIT-TO ( PUBYEAR , 2007 ) OR LIMIT-TO ( PUBYEAR , 2006 ) OR LIMIT-TO ( PUBYEAR , 2005 ) OR LIMIT-TO ( PUBYEAR , 2004 ) OR LIMIT-TO ( PUBYEAR , 2003 ) OR LIMIT-TO ( PUBYEAR , 2002 ) OR LIMIT-TO ( PUBYEAR , 2000 ) ) AND ( LIMIT-TO ( LANGUAGE , "english" ) ) | 175 |

**Web of Science**

| 1 | TS=("academic institut*") | 12,042 |
| --- | --- | --- |
| 2 | TS=("research institut*") | 37,724 |
| 3 | TS=(universit*) | 883,779 |
| 4 | TS=(colleg*) | 347,815 |
| 5 | TS=("post-secondary") | 3,874 |
| 6 | TS=(“higher education”) | 129,402 |
| 7 | TS=(academi*) | 606,422 |
| 8 | TS=(postsecondary) | 5,265 |
| 9 | TS=(student) | 956,688 |
| 10 | #1 OR #2 OR #3 OR #4 OR #5 OR #6 OR #7 OR #8 OR #9 | 2,297,867 |
| 11 | TS=(collaborat*) | 469,936 |
| 12 | TS=(partnership*) | 103,135 |
| 13 | TS=("partnership model*") | 1,084 |
| 14 | TS=("partnership framework*") | 99 |
| 15 | TS=("collaborat* model*") | 3,156 |
| 16 | TS=("model* for collaboration") | 192 |
| 17 | TS=("model* for partnership*") | 38 |
| 18 | TS=("partnership* for collaboration") | 5 |
| 19 | TS=("collaborat* framework*") | 1,164 |
| 20 | TS=(cooperati*) | 419,619 |
| 21 | #11 OR #12 OR #13 OR #14 OR #15 OR #16 OR #17 OR #18 OR #19 OR #20 | 940,104 |
| 22 | TS=("non profit*") | 8,626 |
| 23 | TS=("non government*") | 15,263 |
| 24 | TS=(nonprofit*) | 13,170 |
| 25 | TS=(nongovernment*) | 5,578 |
| 26 | #22 OR #23 OR #24 OR #25 | 40,965 |
| 27 | TS=(healthcare) | 335,417 |
| 28 | TS=("health care") | 447,289 |
| 29 | TS=(hospital) | 1,190,237 |
| 30 | #27 OR #28 OR #29 | 1,778,782 |
| 31 | TS=(technolog*) | 2,472,711 |
| 32 | TS=(simulat*) | 3,940,650 |
| 33 | TS=("simulation based education") | 858 |
| 34 | TS=(innovat*) | 665,143 |
| 35 | TS=(“three dimensional printing”) | 4,005 |
| 36 | #31 OR #32 OR #33 OR #34 OR #35 | 6,590,454 |
| 37 | #10 AND #21 AND #26 AND #30 AND #36 | 69 |
| 38 | #10 AND #21 AND #26 AND #30 AND #36 and English (Languages) and 2023 or 2022 or 2021 or 2020 or 2019 or 2018 or 2017 or 2016 or 2015 or 2014 or 2013 or 2012 or 2011 or 2010 or 2009 or 2008 or 2007 or 2006 or 2005 or 2004 or 2003 or 2002 (Publication Years) | 66 |

**CINAHL**

| 1 | "academic institut*" | 3,104 |
| --- | --- | --- |
| 2 | (MM "Academic Medical Centers") | 9,479 |
| 3 | "research institut*" | 3,487 |
| 4 | universit* | 190,488 |
| 5 | (MH "Colleges and Universities+") | 52,736 |
| 6 | colleg* | 143,025 |
| 7 | (MH "Students, College") | 28,744 |
| 8 | "post secondary" | 773 |
| 9 | postsecondary | 1,105 |
| 10 | S1 OR S2 OR S3 OR S4 OR S5 OR S6 OR S7 OR S8 OR S9 | 315,257 |
| 11 | collaborat* | 131,432 |
| 12 | (MM "Collaboration") | 18,759 |
| 13 | partnership* | 36,982 |
| 14 | "partnership model*" | 469 |
| 15 | "partnership framework*" | 25 |
| 16 | "collaborat* model*" | 970 |
| 17 | "model* for collaboration" | 261 |
| 18 | "model* for partnership*" | 97 |
| 19 | "partnership* for collaboration" | 138 |
| 20 | "collaborat* framework*" | 102 |
| 21 | (MH "Cooperative Behavior") | 9,010 |
| 22 | (MH "Interinstitutional Relations") | 10,188 |
| 23 | S11 OR S12 OR S13 OR S14 OR S15 OR S16 OR S17 OR S18 OR S19 OR S20 OR S21 OR S22 | 168,958 |
| 24 | "non profit*" | 2,056 |
| 25 | (MH "Organizations, Nonprofit") | 10,783 |
| 26 | "non government*" | 2,786 |
| 27 | nonprofit* | 14,097 |
| 28 | nongovernment* | 1,208 |
| 29 | S24 OR S25 OR S26 OR S27 OR S28 | 19,386 |
| 30 | (MH "Health Care Delivery") | 64,138 |
| 31 | healthcare | 768,373 |
| 32 | "health care" | 605,947 |
| 33 | hospital | 582,042 |
| 34 | (MH "Hospitals") | 68,346 |
| 35 | S30 OR S31 OR S32 OR S33 OR S34 | 1,215,746 |
| 36 | (MH "Technology") | 21,362 |
| 37 | technolog* | 217,407 |
| 38 | simulat* | 83,083 |
| 39 | (MH "Simulations+") | 48,262 |
| 40 | "simulation-based education" | 405 |
| 41 | innovat* | 82,631 |
| 42 | (MH "Diffusion of Innovation") | 18,174 |
| 43 | (MH "Printing, Three-Dimensional") | 2,526 |
| 44 | S36 OR S37 OR S38 OR S39 OR S40 OR S41 OR S42 OR S43 | 369,877 |
| 45 | S10 AND S23 AND S29 AND S35 AND S44 | 37 |
